# B-cells and regulatory T-cells in the microenvironment of HER2+ breast cancer are associated with decreased survival: a real-world analysis of women with HER2+ metastatic breast cancer

**DOI:** 10.1186/s13058-023-01717-1

**Published:** 2023-10-04

**Authors:** Tessa G. Steenbruggen, Denise M. Wolf, Michael J. Campbell, Joyce Sanders, Sten Cornelissen, Bram Thijssen, Roberto A. Salgado, Christina Yau, Nick O-Grady, Amrita Basu, Rajith Bhaskaran, Lorenza Mittempergher, Gillian L. Hirst, Jean-Philippe Coppe, Marleen Kok, Gabe S. Sonke, Laura J. van ‘t Veer, Hugo M. Horlings

**Affiliations:** 1 Department of Medical Oncology, The Netherlands Cancer Institute, 1066 CX Amsterdam, North Holland The Netherlands; 2 Department of Laboratory Medicine, University of California San Francisco, San Francisco, CA 94115 USA; 3 Department of Surgery, University of California San Francisco, San Francisco, CA 94115 USA; 4 Department of Pathology, The Netherlands Cancer Institute, 1066 CX Amsterdam, North Holland The Netherlands; 5 Core Facility Molecular Pathology and Biobanking, The Netherlands Cancer Institute, 1066 CX Amsterdam, North Holland The Netherlands; 6 Department of Molecular Carcinogenesis, The Netherlands Cancer Institute, 1066 CX Amsterdam, North Holland The Netherlands; 7 Department of Pathology, GZA-ZNA Hospitals, 2020 Antwerp, Belgium; 8 Division of Research, Peter Mac Callum Cancer Centre, Melbourne, VIC 3000 Australia; 9 Research and Development, Agendia N.V, 1043 NT Amsterdam, North Holland The Netherlands; 10 Division of Tumor Biology and Immunology, The Netherlands Cancer Institute, 1066 CX Amsterdam, North Holland The Netherlands; 11 Department of Clinical Oncology, University of Amsterdam, 1012 WX Amsterdam, North Holland The Netherlands

**Keywords:** HER2-positive, Metastatic breast cancer, Tumor immune microenvironment, Multiplex immunofluorescence, Spatial composition, Gene expression

## Abstract

**Background:**

Despite major improvements in treatment of HER2-positive metastatic breast cancer (MBC), only few patients achieve complete remission and remain progression free for a prolonged time. The tumor immune microenvironment plays an important role in the response to treatment in HER2-positive breast cancer and could contain valuable prognostic information. Detailed information on the cancer-immune cell interactions in HER2-positive MBC is however still lacking. By characterizing the tumor immune microenvironment in patients with HER2-positive MBC, we aimed to get a better understanding why overall survival (OS) differs so widely and which alternative treatment approaches may improve outcome.

**Methods:**

We included all patients with HER2-positive MBC who were treated with trastuzumab-based palliative therapy in the Netherlands Cancer Institute between 2000 and 2014 and for whom pre-treatment tissue from the primary tumor or from metastases was available. Infiltrating immune cells and their spatial relationships to one another and to tumor cells were characterized by immunohistochemistry and multiplex immunofluorescence. We also evaluated immune signatures and other key pathways using next-generation RNA-sequencing data. With nine years median follow-up from initial diagnosis of MBC, we investigated the association between tumor and immune characteristics and outcome.

**Results:**

A total of 124 patients with 147 samples were included and evaluated. The different technologies showed high correlations between each other. T-cells were less prevalent in metastases compared to primary tumors, whereas B-cells and regulatory T-cells (Tregs) were comparable between primary tumors and metastases. Stromal tumor-infiltrating lymphocytes in general were not associated with OS. The infiltration of B-cells and Tregs in the primary tumor was associated with unfavorable OS. Four signatures classifying the extracellular matrix of primary tumors showed differential survival in the population as a whole.

**Conclusions:**

In a real-world cohort of 124 patients with HER2-positive MBC, B-cells, and Tregs in primary tumors are associated with unfavorable survival. With this paper, we provide a comprehensive insight in the tumor immune microenvironment that could guide further research into development of novel immunomodulatory strategies.

**Graphical Abstract:**

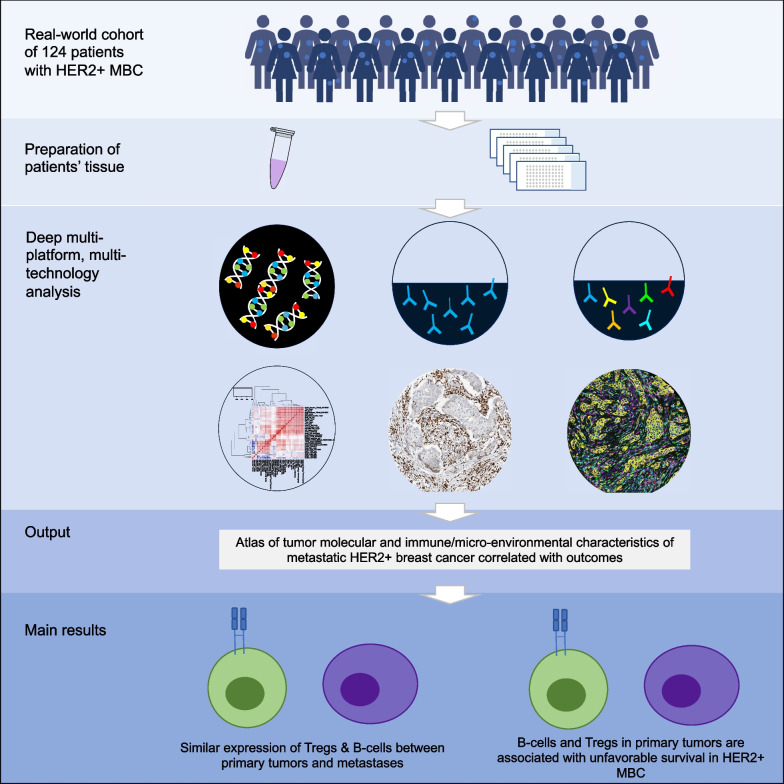

**Supplementary Information:**

The online version contains supplementary material available at 10.1186/s13058-023-01717-1.

## Background

Outcome for patients with human epidermal growth factor receptor 2 (HER2)-positive metastatic breast cancer (MBC) has dramatically improved since the introduction of trastuzumab. More recently, the addition of pertuzumab, ado-trastuzumab emtansine (TDM-1), trastuzumab-deruxtecan, tucatinib, neratinib, and margetuximab have further increased outcome in these patients [1–6]. Follow-up of the CLEOPATRA study showed that a small group of patients experience long-term progression-free survival [1]. We and others have previously shown in real-world cohorts that achieving radiological complete remission (rCR) is strongly associated with improved overall survival (OS) in patients with HER2-positive MBC [7, 8]. Strikingly, survival ranges from a few months to several years and sometimes even decades. Insight into tumor characteristics and the immune microenvironment of primary and metastatic tumor samples from patients with MBC could provide insight into the underlying processes of such variable outcomes and potentially aid in personalization of treatment and ultimately long-term survival for more patients [9].

Within the tumor immune microenvironment both protumor and antitumor cells play a role, such as protumor neutrophils promoting metastases or regulatory T-cells allowing tumor proliferation and on the other side CD8+ T-cells and natural killer (NK) cells that elicit favorable anti-tumor immune responses [10]. The tumor immune microenvironment may be of particular importance in HER2-positive breast cancer as HER2 is a natural antigen and the response to HER2-targeted therapies is partly based on both the innate immune system via antibody-dependent cell cytotoxicity (ADCC) as the adaptive immune system, by means of NK-cell activation [11, 12]. Preclinical studies have shown that activation of the immune system, in particular NK-cells, is necessary for trastuzumab efficacy [13, 14]. Therefore the cellular composition of the tumor immune microenvironment may be associated with outcome in HER2-positive MBC, as has been shown for triple-negative MBC [5].

In patients with HER2-positive MBC, the prognostic value of stromal tumor infiltrating lymphocytes (sTILs) showed conflicting results in retrospective analyses of several studies and a retrospective series of patients mainly evaluating sTILs in quantitative manner [15–18]. In-depth characterization of immune cells may have key prognostic value and increase our understanding of the interaction between tumor and microenvironment [19]. It may also provide clues for development of immune modulating agents that can be combined with anti-HER2 treatment. In patients with early breast cancer, multi-omics features and single-cell pathology data were highly correlated with outcome [20, 21].

In this exploratory analysis we use a combination of next-generation RNA sequencing, multiplex immunofluorescence (mIF) multispectral analysis for spatial composition evaluation, and immunohistochemistry (IHC) to characterize the tumor immune microenvironment of 108 pre-treatment primary tumor samples and 39 samples of metastases of 124 patients with HER2-positive MBC, including 15 paired samples. Next, we evaluate if immune traits associate with rCR and survival in a real-world cohort of patients with long-term follow up.

## Materials and methods

### Clinical data

We included all patients (*n* = 135) with histologically proven HER2-positive MBC who were treated with trastuzumab-based palliative therapy in the Netherlands Cancer Institute between January 2000 and January 2014 and for whom pre-treatment tissue from the primary tumor or from metastases was available (Additional file 2: Fig. S1). Patient and tumor characteristics were extracted from the medical records by two reviewers. Details on extraction of clinical data have been described previously [7] For patients referred to the Netherlands Cancer Institute, tissue was collected via linkage with the nationwide network and registry of histo- and cytopathology in the Netherlands (PALGA Foundation). The Institutional Review Board of the NKI approved this study.

### Next-generation RNA sequencing and signatures

RNA was isolated from formalin-fixed paraffin-embedded (FFPE) samples containing at least 30% tumor cells, located close to infiltrating immune cells. More details are provided in Additional file 1: Supplementary Materials and Methods.

70-gene high versus low-risk and 80-gene subtypes (i.e., Luminal, HER2 or Basal-type) were generated by Agendia using the same methodology as previously translated, calibrated and validated next-generation sequencing read-out from 70-gene and 80-gene micro-array tests [22–24] More details can be found in Additional file 1: Supplementary Materials and Methods.

Thirty-two published signatures were selected for evaluation, including immune-related [25–34], extracellular matrix (ECM)-related [35], proliferation-related [36], and estrogen receptor (ER) and HER2 gene expression signatures [34]. Immune signatures represent T-cell and B-cell signaling, macrophage and dendritic cells, macrophage-to-T-cell (CD8-to-CD68) ratio, programmed death 1 (PD1)/programmed death ligand 1 (PDL1) signaling, interferon signaling, interleukin/cytokine signatures, and transforming growth factor beta (TGF-β) signaling (Additional file 3: Table S1). Data were mean-centered prior to signature evaluation.

### Scoring of stromal tumor-infiltrating lymphocytes

Three experienced pathologists (JS, HH, RS) scored hematoxylin and eosin (H&E)-stained whole sections of 124 patients for sTILs using the method that was standardized by the international TILs working group and externally validated [37]. In brief, sTILs were scored as the percentage of stroma, interpreted visually in the context of a reference image. The sTILs evaluation was concordant between the three pathologists (data not shown). In case of > 10% difference a consensus score was reached.

For all patients sTILs were evaluated, IHC (ER, progesterone receptor [PR], HER2, androgen receptor [AR], CD3, CD8, CD20, CD56, CD68, and PDL1) was evaluable for 123 patients (110 samples of primary tumors and 39 samples of metastases, of which 26 were pairs), mIF panels could be analyzed for 103 patients (99 samples of primary tumors and 19 samples of metastases, of which 15 were pairs) and we were able to obtain RNA sequencing data of sufficient quality for 97 patients (91 samples of primary tumors and 21 samples of metastases, of which 15 were pairs).

### Multispectral immunofluorescence

We used two mIF panels to evaluate the expression of CD3, CD20, FoxP3, CK, and Ki-67 (Panel 1) and the expression of CD3, CD8, CD68, PD1, PDL1, and CK (Panel 2; example Additional file 2: Fig. S2) in the tumor microenvironment. Specific antibody clones are listed in Additional file 3: Table S2.

### Spatial distribution analysis (colocalization)

Spatial distribution analysis was performed on the cell segmentation data in the R environment (R version 3.6.1) using the *spatstat* package for analyzing spatial point patterns [38]. We applied the Morisita-Horn index [39] to the cell phenotype data to quantify spatial colocalization of cancer cells and immune cells as well as immune cells with other immune cells. Each mIF image was virtually divided into non-overlapping squares of 100 µm × 100 µm and the number of cancer cells and immune cells (of each phenotype) within each square were counted. Morisita-Horn’s similarity index was then calculated for various pairs of cell types (e.g., Tumor cells and T-cells, or T-cells and macrophages). The Morisita-Horn index ranges from 0, indicating no colocalization of the two cell types (e.g., each square contains only tumor cells or immune cells), to 1, where the two cell types are highly colocalized (e.g., each square contains an equal number of tumor cells and immune cells) (Additional file 2: Fig. S3A).

We also evaluated the spatial relationships of different cells in the tumor microenvironment using the nearest neighbor distance distribution function G(r) resulting in the Spatial Proximity Score (SPS) and the Ecoscore (Additional file 2: Fig. S3B, C). Details on calculation of both scores as well as details on tissue microarray (TMA) construction, mIF, IHC and expression scoring can be found in Additional file 1: Supplementary Materials and Methods.

### Statistical analyses

Patient characteristics are presented as medians with IQR for continuous variables and as percentages for categorical variables. The primary endpoint was OS, defined as date of diagnosis of MBC until death from any cause [40]. For patients last known to be alive, OS data were censored at the time of last follow-up visit. Follow-up time was calculated with the reverse Kaplan–Meier method. We used Cox proportional hazards modeling to assess the correlation of variables of interest—gene signatures and immune biomarkers—with OS, adjusted for ER status and rCR. Additionally, we explored expression of biomarkers between and within patients achieving rCR and patients who did not achieve rCR using frequency plots. The association between biomarkers and rCR was assessed using linear regression models, adjusted for ER status. Hazard ratios (HR) and odds ratios (OR) (per unit increase) are reported with their corresponding likelihood ratio (LR) *P* value.

Correlations between signatures and cell phenotypes were explored using Spearman rho’s correlations coefficient. Correlation of sTILs and clinicopathological characteristics were compared using Spearman correlation for continuous variables, Mann–Whitney U tests for binary variables, and the Kruskal–Wallis H test for variables with more than two groups. Correlation figures are prepared using hierarchical clustering. The predictive value of sTILs was evaluated considering sTILs as a continuous variable.

All statistical tests were two-sided and considered statistically significant when *P* < 0.05. In this hypothesis generating, exploratory analysis we report both uncorrected (main manuscript and figures) and multiple hypothesis corrected (available in supplementary results file; Additional file 4: Results Table) *P* values, the latter adjusted using the Benjamini–Hochberg method [41]. Because most (immune) variables are highly correlated and subset sizes are small, we used results from uncorrected *P* values (LR *P* < 0.05) to shape the narrative. All calculations were performed using R version 3.6.1.

## Results

### Clinical characteristics and outcomes

We collected data from patients treated with trastuzumab for HER2-positive MBC in The Netherlands Cancer Institute between 2000 and 2014, described in our previously published study [7], for whom tissue samples were available and sufficient for further evaluation. This resulted in a study-cohort of 124 patients. Thirty patients (24%) were diagnosed with de novo metastases. In patients with recurrent MBC, median time until metastases was 38 months (interquartile range [IQR] 22–60). Fifty-five percent of patients had ER-positive/HER2-positive breast cancer. Most patients had skeletal metastases, followed by lung metastases and distant lymph node metastases. More clinical and treatment characteristics are shown in Table 1. After median follow-up of 8.9 years (IQR 8.3-not reached), 104 patients had died, 102 from MBC. Twenty (16%) patients achieved rCR, of whom 5 remained in remission until last follow-up and 10 patients are still alive at last follow-up (14+ years). We have previously shown that achieving rCR is strongly associated with long-term OS [7]. We therefore performed survival association analyses in all patients, adjusting for rCR and separately in patients achieving and not achieving rCR.

**Table 1:** Baseline clinical and pathological characteristics

	Patients with evaluable tissue (*n* = 124)	
	*n*	%
Age at diagnosis MBC, no (%)		
≤ 50 years	60	48
> 50 years	64	52
Time till MBC, no (%)		
de novo MBC	30	24
≤ 36 months	45	36
> 36 months	49	40
ER-status at diagnosis, no. (%)		
ER-positive	68	55
ER-negative	56	45
PR-status at diagnosis*		
PR-positive	45	36
PR-negative	76	61
HER2 IHC*		
1+	1	1
2+	12	10
3+	107	86
Grade primary tumor*		
Grade 1 or 2	34	27
Grade 3	66	53
Grade unknown	24	19
Single-organ metastases, no. (%)		
Metastases in a single organ	72	58
Metastases in more organs	52	42
Oligo-metastases (≤ 3 metastases), no. (%)		
Oligo-metastases†	39	31
Multiple metastases	85	69
Location of metastases at diagnosis MBC, no. (%)		
Bone	60	48
Liver	44	35
Lymph nodes	45	36
Lung	36	29
Skin	10	8
CNS	14	11
Prior neoadjuvant/adjuvant trastuzumab, no. (%)		
Yes	29	23
No	95	77
Moment first trastuzumab for MBC, no. (%)		
Trastuzumab received in 1st line	95	77
Trastuzumab received in 2nd line	29	23
Received pertuzumab for MBC, no (%)		
Yes	0	0
No	124	100

*CNS*—Central nervous system

*Number of patients with unknown data are not shown

†23 of the 39 patients with oligometastases received local (ablative) treatment of their metastases

### Molecular-pathological evaluation

**Fig. 1:**
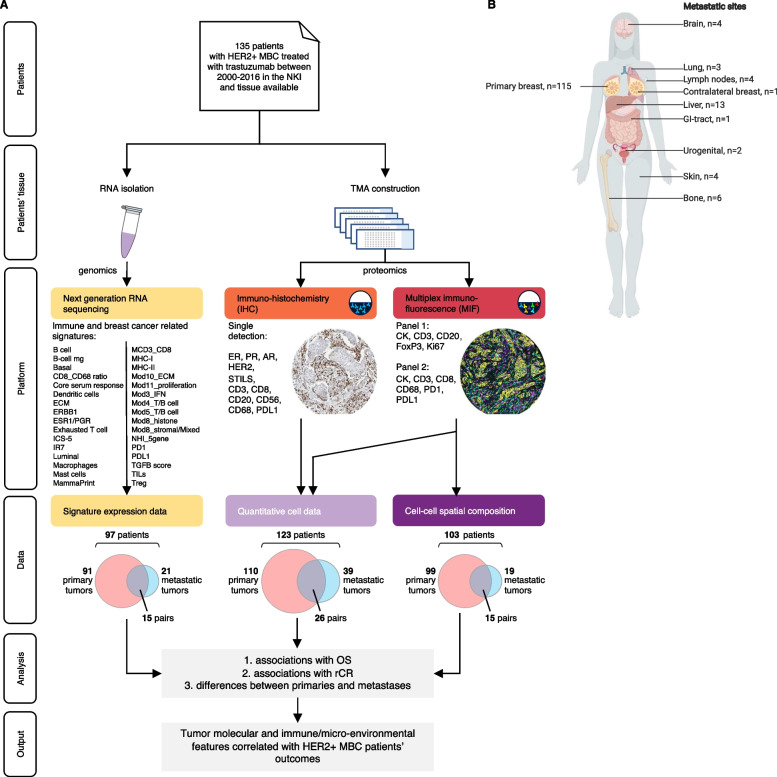
Study overview. **A** shows the workflow of patient data and tissue collection to the evaluation with different technologies, i.e., RNA sequencing (yellow), immunohistochemistry (orange) and multiplex immunofluorescence (red). The different platforms provided signatures expression data (yellow), quantitative cell data (lavender) and cell-to-cell spatial composition data (purple). For 97 patients, signature expression data was available, this included 91 primary tumor samples and 21 metastasis samples. For 15 patients a sample of the primary tumor and of a metastasis was available. For 123 patients quantitative cell data was available, this included 110 primary tumor samples and 39 metastasis samples. For 26 patients a sample of the primary tumor and of a metastasis was available. For 103 patients cell-to-cell spatial composition data was available, this included 99 primary tumor samples and 19 metastasis samples. For 15 patients a sample of the primary tumor and of a metastasis was available. We evaluated associations with OS and rCR as well as differences between primary tumors and metastases. This led to thorough overview of tumor molecular and immune micro-environmental features correlated with HER2-positive MBC patients’ outcomes. **B** is an overview of sample sites. The number of samples is indicated. Credit: Created with BioRender (https://biorender.com/)

To characterize the tumor and its microenvironment in detail, we examined formalin-fixed, paraffin-embedded tumor tissue samples with three different technologies (next-generation RNA sequencing, IHC, and mIF; Fig. 1A). A consort-flow diagram, including number of primary tumor samples and metastasis samples, is shown in Additional file 2: Fig. S1. The location of the metastatic samples is shown in Fig. 1B.

### Different technologies are congruent in reporting biology

Results from the different technologies were each summarized into scores, including gene expression signatures from RNA sequencing data, summary statistics of the immune cell infiltration and cell-to-cell spatial composition scores of various cell types calculated using the Morisita-Horn index [39] (see Additional file 2: Fig. S3A for details). The resulting scores showed high correlations between the different technologies (i.e., RNA sequencing, mIF, and IHC; Additional file 2: Fig. S4). For example, HER2 IHC scores and SISH scores clustered together with the *ERBB2*-amplicon signature and the 80-gene HER2 score. ER and PR expression measured by IHC clustered together with *ESR1/PGR* signature as well as with the 80-gene Luminal score and 70-gene index. T-cells and B-cells measured with IHC clustered together with T-cells and B-cells measured by mIF. In addition, PDL1 expression as measured by IHC clustered together with the PDL1 expression measured by mIF and PDL1 data clustered together with the exhausted T-cell signature. Last, samples from the primary tumor and metastases of the same patient grouped together in an unsupervised cluster diagram (Additional file 2: Fig. S5).

### ER and PR positivity is associated with better outcome

**Fig. 2:**
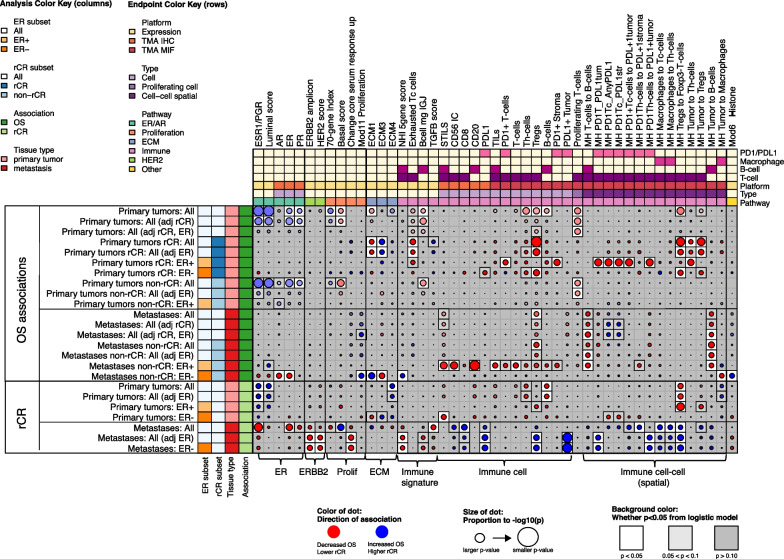
Association dot plot all data technologies with OS and rCR. In this dot plot all significant associations in at least one analysis are shown. Blue dots indicate increased overall survival or higher likelihood of radiological complete response. Red dots indicate decreased overall survival or lower likelihood of radiological complete response. The size of the dot is proportional to the *P* value with larger dots indicating a smaller *P* value. The background color is white for *P* < 0.05, light gray for *P* > 0.05 and < 0.10 and gray for *P* ≥ 0.10. *P* values shown are not adjusted for multiple testing. Data from both primary tumors and metastases are analyzed, indicated on the left and by red and pink boxes, respectively. Analyses in subgroups are indicated by colors: hormone-receptor subgroup analyses (orange) and radiological complete response subgroup (blue). Analyses adjusted for radiological complete response are indicated by blue boxes as well. Dark green boxes indicate associations with overall survival. Light green boxes indicate association with radiological complete response. Overarching pathways are indicated by colors on top. Platform can be gene expression (yellow), TMA IHC (orange), or TMA MIF (red). Type refers to whether TMA measurements are of an individual cell type (lavender), proliferating cells (purple), or cell–cell spatial relationships (dark purple)

We evaluated how classical breast cancer biomarkers and subtypes; i.e., ER, PR, HER2, and basal phenotypes evaluated by IHC and 80-gene score were associated with OS and rCR. Positive ER and PR status in the primary tumor as measured by IHC correlated with better OS, as did higher expression of *ESR1/PGR* and the continuous Luminal index. The *ESR1/PGR* signature remained significantly associated with OS after adjusting for achieving rCR (Fig. 2 and Additional file 2: Fig. S6A, B). *ESR1/PGR* expression, ER and PR IHC, and the Luminal index in the primary tumor were positively associated with rCR in all patients as well (Fig. 2 and Additional file 2: Fig. S6C).

In addition, we evaluated whether the prognostic *70-gene signature* was associated with outcome in MBC. As expected, almost all (*n* = 85, 83%) primary tumors were classified as 70-gene high-risk. The continuous 70-gene index was associated with better OS, HR 0.70, *P* = 0.027 (Fig. 2).

**Fig. 3:**
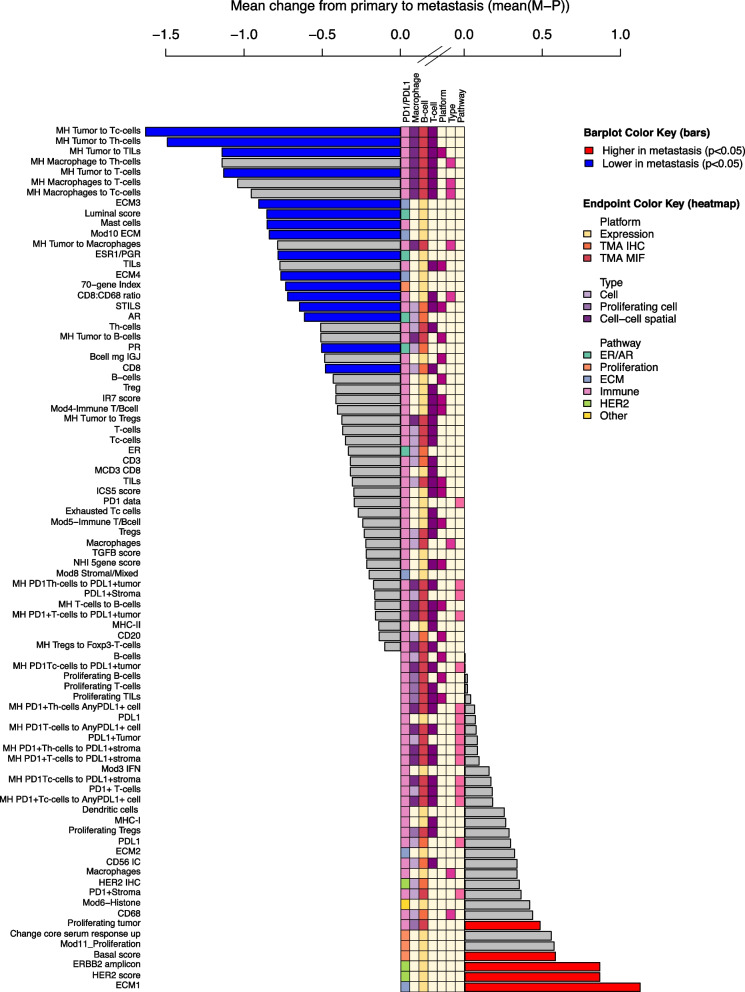
HER2 expression, extracellular matrix signatures and spatial relationships differ between primary tumors and metastases. Figure 3 shows the mean difference in expression between primary tumors and metastases. Bars to the left indicate lower expression in metastases compared to the primary tumors. Blue bars indicate that the difference is statistically significantly lower in metastases (*P* < 0.05). Bars to the right indicate higher expression in metastases compared to the primary tumors. Red bars indicate that the difference is statistically significantly higher in metastases (*P* < 0.05). Overarching pathways are indicated by colors on top. Platform can be gene expression (yellow), TMA IHC (orange), or TMA MIF (red). Type refers to whether TMA measurements are of an individual cell type (lavender), proliferating cells (purple), or cell–cell spatial relationships (dark purple)

We also evaluated the relative levels of receptor subtype-related signals in paired primary and metastatic tumors from 26 patients. *ESR1/PGR* signature, the Luminal score, and IHC ER, PR and AR expression were lower in metastases compared to primary tumors (Fig. 3, blue bars, turquoise pathway boxes). In contrast, the HER2 score, expression levels of the *ERBB2* amplicon signature, and the Basal score were higher in metastases (Fig. 3, red bars, green pathway boxes). As these results highlight the important influence of ER status in HER2-positive breast cancer, we evaluated all further analyses with and without adjusting for ER status as well as per HER2-positive/ER-positive and HER2-positive/ER-negative subgroups.

### Stromal tumor infiltrating lymphocytes are not associated with outcomes

The median sTILs percentage in the primary tumor was 7% (IQR 3–30%) (Additional file 2: Fig. S7A). sTILs were not statistically significantly associated with OS (HR 1.08, *P* = 0.454) nor with rCR (OR 0.66, *P* = 0.167). Stromal TILs percentages were similar in HER2-positive/ER-positive and HER2-positive/ER-negative tumors and not associated with outcome in either subgroup. A longer interval (> 36 months) between primary breast cancer and metastatic recurrence was associated with higher sTILs percentages and higher clinical nodal stage was associated with lower sTILs values (Additional file 3: Table S3). Stromal TILs percentages were similar between patients with oligometastases (3 or less metastases) and patients having more than three metastases. The median sTILs percentage in metastases was 1% (IQR 1–7%), which was lower than in the primary tumor,* P* < 0.001. Lung and lymph node samples had the highest percentages of sTILs (Additional file 2: Fig. S7A). Also, in paired samples, presence of sTILs was significantly lower in metastatic samples than in the primary tumor. The presence of sTILs in metastases was not associated with OS, nor with rCR (Additional file 2: Fig. S7B–D).

Next, we characterized sTILs using mIF and IHC to evaluate different immune cells in the tumor immune microenvironment.

### Infiltration of Tregs and exhausted T-cells are associated with unfavorable survival

We scored patient samples for the following T-cell subsets: cytotoxic T-cells (CD3+, CD8+), helper T-cells (CD3+, CD8−), regulatory T-cells (CD3+, FoxP3+, CD8−), FoxP3-negative T-cells (CD3+, FoxP3−), and programmed cell death protein 1 (PD1) + T-cells (CD3+, PD1+) using mIF; as well as NK-cells (CD56+) using IHC. In addition, we evaluated several T-cell related gene expression signatures including *exhausted T-cell, regulatory T-cell,* and *MCD3_CD8* (see Additional file 3: Table S1 for signature details). Taken together, in our cohort two T-cell subsets were associated with poor outcomes: regulatory T-cells (Tregs) and exhausted T-cells.

**Fig. 4:**
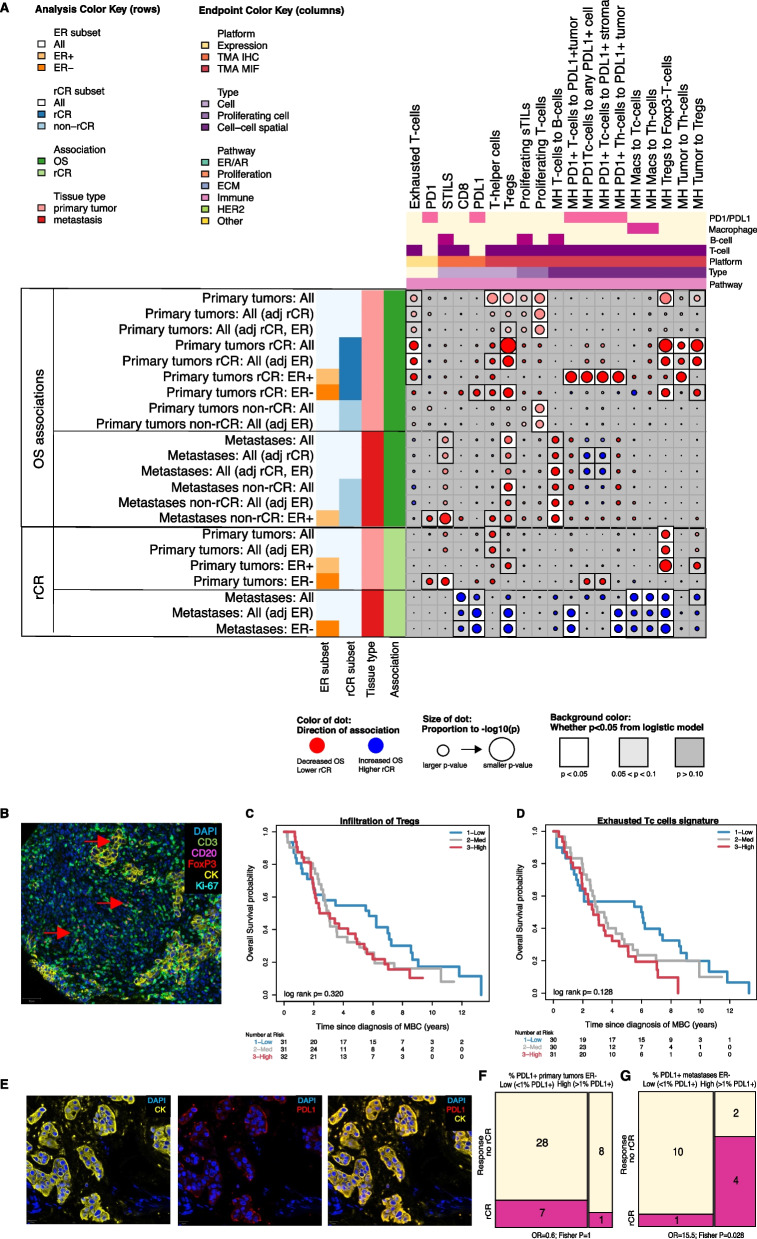
Infiltration of Tregs and exhausted T-cells are associated with unfavorable outcomes. **A** is a close-up dot plot of Fig. 2 focused on all significant associations between T-cell related expression with either overall survival or radiological complete remission in at least one analysis. Blue dots indicate increased overall survival or higher likelihood of radiological complete response. Red dots indicate decreased overall survival or lower likelihood of radiological complete response. The size of the dot is proportional to the P value with larger dots indicating a smaller P value. The background color is white for *P* < 0.05, light gray for *P* > 0.05 and < 0.10 and gray for *P* ≥ 0.10. P values shown are not adjusted for multiple testing. **B** is a multiplex immunofluorescence image showing FoxP3-positive T cells (Tregs; red-colored cells) and FoxP3-negative T cells (green-colored cells), indicated by red arrows. **C** shows the overall survival probability according to infiltration of regulatory T-cells (Tregs) split in tertiles. **D** shows the overall survival probability according to the *exhausted T-cell signature* split in tertiles. **E** is a multiplex immunofluorescence image showing tumor cells (yellow cells), PDL1-positive tumor cells (red cells) and a merged image of PDL1-positive tumor cells. **F** shows PDL1 expression (low vs high) in ER- primary tumors in radiological complete response (pink) and no radiological complete response (yellow). **G** shows PDL1 expression (low vs high) in ER-negative metastases in radiological complete response (pink) and no radiological complete response (yellow)

More infiltration of Tregs was associated with decreased OS in all patients and in rCR-subgroups (Fig. 4A, C). Also, greater colocalization of Tregs and Foxp3-negative T-cells and of Tregs and tumor cells was associated with decreased OS (Fig. 4A and example 4B). Colocalization of Tregs and Foxp3-negative T-cells in the primary tumor was also statistically significantly negatively associated with rCR (Fig. 4A).

Of the other measured T-cells, helper T-cells (CD3+/CD8−) in primary tumors were associated with unfavorable OS in all patients (Fig. 4A).

When comparing infiltration of T-cells in metastatic samples to primary tumors, we found that there were statistically significantly fewer cytotoxic T-cells, helper T-cells, and FoxP3-negative T-cells in metastases (Fig. 3, blue bars). Infiltration of Tregs was comparable between metastases and primary tumors. Infiltration of Tregs in metastases was associated with unfavorable survival similar to expression in primary tumors. In contrast, the infiltration of more Tregs in ER-negative metastases was correlated with a higher chance of achieving rCR (Fig. 4A).

Higher expression of the *exhausted T-cell* signature in primary tumors showed a trend toward unfavorable OS in all patients and was associated with unfavorable OS in the rCR-subgroup (Fig. 4A, D). PD1 expression was not associated with OS nor rCR in the population as a whole, but did associate with OS in the rCR ER-positive subgroup. Other T-cell signatures did not show statistically significant associations (Additional file 4: Results Table).

The expression of PDL1 on tumor cells is shown in Fig. 4G. PDL1 expression was not statistically significantly lower in metastases compared to primary tumors (Fig. 3). In HER2+/ER-negative metastases but not HER2+/ER-negative primary tumors, higher PDL1 expression on tumor cells was associated with achieving rCR (Fig. 4F, G). Higher levels of colocalization of PD1-positive T-cells and PDL1-positive tumor cells in metastases was also associated with rCR. The only T-cell or PD1/PDL1-related signal to hint at an association with improved OS was the colocalization of PD1-positive T-cells and PDL1-positive tumor cells in metastatic samples, which trended toward better OS after adjusting for rCR (Fig. 4A, rows 11–12). All other T/PD1/PDL1 signals in primary or metastatic samples associated with decreased OS, if at all.

**Fig. 5:**
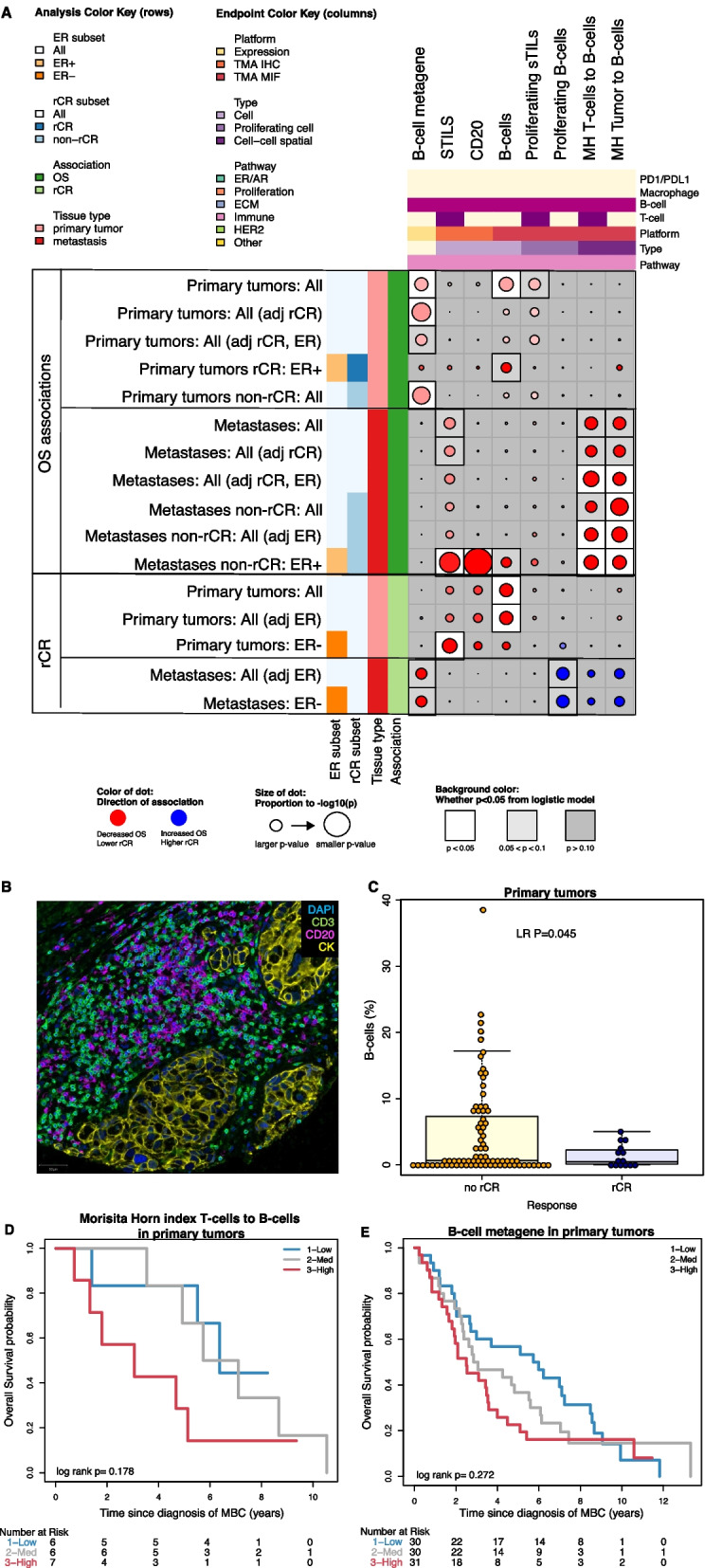
B-cell infiltrates are associated with unfavorable outcomes. **A** is a close-up dot plot of Fig. 2 focused on all significant associations between B-cell related expression with either overall survival or radiological complete remission in at least one analysis. Blue dots indicate increased overall survival or higher likelihood of radiological complete response. Red dots indicate decreased overall survival or lower likelihood of radiological complete response. The size of the dot is proportional to the P value with larger dots indicating a smaller *P* value. The background color is white for *P* < 0.05, light gray for *P* > 0.05 and < 0.10 and gray for *P* ≥ 0.10. *P* values shown are not adjusted for multiple testing. **B** is a multiplex immunofluorescence image showing B-cells (CD20 + ; magneta-colored cells) and T-cells (CD3 + ; green-colored cells) as well as tumor cells (CK + , yellow-colored cells). **C** shows infiltration of B-cells in primary tumors in patients with radiological complete response (blue) and patients with no radiological complete response (yellow). **D** shows the overall survival probability according to B-cells and T-cells colocalization intensity measured with the Morisita-Horn index in primary tumors split in tertiles. **E** shows the overall survival probability according to expression of the *B-cell metagene signature* in primary tumors split in tertiles

### B-cell infiltrates are associated with unfavorable outcomes

Another important subset of infiltrating lymphocytes are B-cells, known to highly interact with other immune cells in the tumor microenvironment. We scored CD20 expression (using both IHC and mIF) to measure the infiltration of B-cells in primary and metastatic tumor samples. In addition, we used two B-cell specific signatures to evaluate impact of B-cells on outcomes, i.e., a *non-cancer specific B-cell signature* and *B-cell metagene signature*, and several T/B-cell-related signatures (Additional file 3: Table S1).

Multiplex analysis showed that the infiltration of B-cells in primary tumors was associated with unfavorable OS and lower likelihood of rCR (Fig. 5A, C).

Levels of B-cells as measured by mIF was comparable between primary tumors and metastases (Fig. 3) and not statistically associated with outcome in all patients. Analyzing the spatial relationships between B-cells and other cells, we found that colocalization of B-cells and tumor cells or T-cells was associated with unfavorable survival (Fig. 5B, D).

The *B-cell metagene* signature was associated with unfavorable OS in all patients (Fig. 5A, E), although the effect was not statistically significant after adjusting for rCR and ER-status.

### Signatures classifying the extracellular matrix of primary tumors showed differential survival

**Fig. 6:**
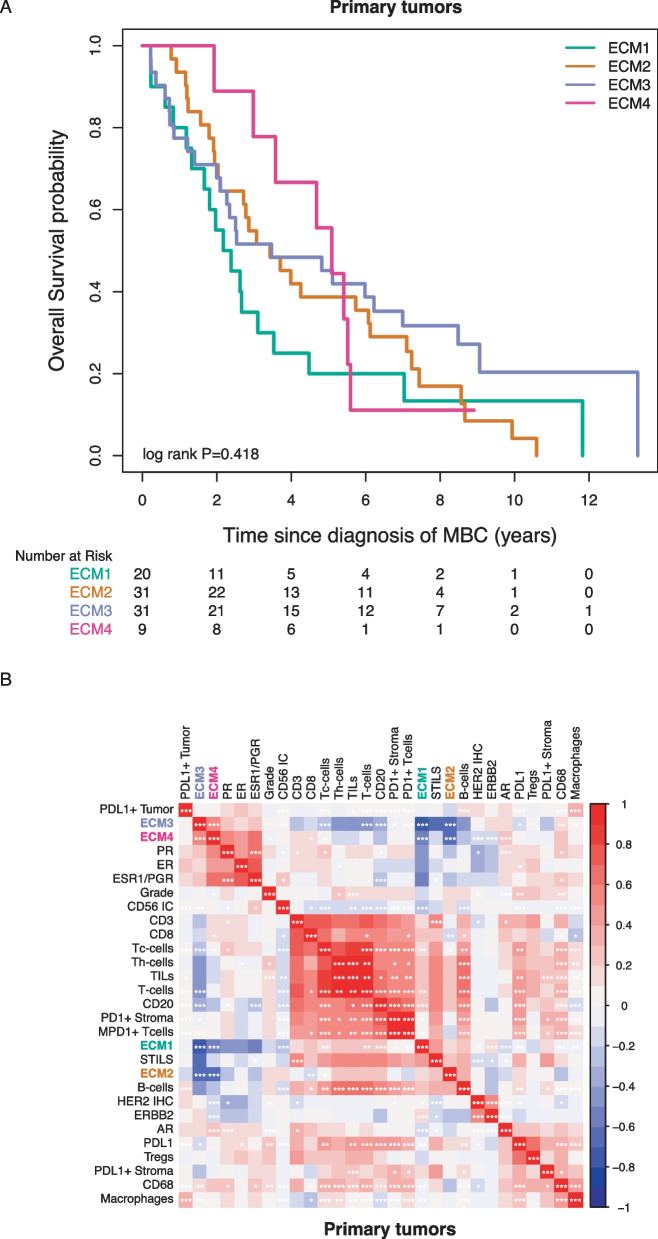
Signatures classifying the extracellular matrix of primary tumors showed differential survival. **A** shows the overall survival probability according to expression of the ECM1, ECM2, ECM3, ECM4 signature in primary tumors. **B** is a correlation heat map of tumor characteristics, immune cells and ECM signatures in primary tumors. The color relates to the direction of the correlation (red = positive correlation, blue = negative correlation); color intensity relates to the strength of the correlation. Significant correlations are indicated by *, **, *** for Spearman’s *P* < 0.05, < 0.01, or 0.001 respectively

The composition of the ECM in terms of the amount and density of collagen and fibrin may determine whether infiltration of immune cells is possible. Four signatures classifying the ECM of primary tumors showed differential survival in the population as a whole (Fig. 6A). The ECM4 signature, identifying a mainly “inflammatory ECM”, as a continuous variable trended toward association with better OS in all patients and the ECM1 signature (“highly-vascularized ECM”) was significantly associated with unfavorable survival in all patients (Fig. 2).

Expression of ECM3 (“dense ECM”) and ECM4 was lower in metastases and in contrast expression of ECM1 was higher in metastases compared to primary tumors (Fig. 3, blue pathway boxes).

ECM1 expression in primary tumors also correlated with ER- and PR-negativity in primary tumors. ER-, PR-, and AR-positivity were correlated with expression of the prognostically most favorable ECM4 signature (Fig. 6B). Higher expression of the prognostically favorable ECM3 signature correlated with less infiltration of sTILs in primary tumors whereas higher expression of prognostically unfavorable ECM1 signature was associated with more infiltration of sTILs in primary tumors (Fig. 6B). The ECM2 signature, with overexpression of metabolic pathways in the ECM, was not associated with outcome in our cohort.

## Discussion

We characterized the tumor immune microenvironment in 147 samples of 124 real-world patients with HER2-positive MBC and in exploratory analysis evaluated whether the infiltration of specific immune cells was associated with rCR and with OS. In our study with long-term follow-up of more than 9 years, we have shown that the infiltration of B-cells, regulatory T-cells, and exhausted T-cells are associated with unfavorable outcome. Additionally, the extracellular matrix signature ECM1 (high vascularization in the ECM), is associated with more infiltration of sTILs and negatively associated with OS. Last, more PDL1-positive cells in metastases was associated with higher likelihood of rCR and trended toward better OS. Below we will discuss our most important findings on infiltration of specific immune cells and how these insights could serve the evaluation and development of novel immunomodulatory strategies.

In our cohort of patients with HER2-positive MBC, the presence of overall sTILs was not associated with OS nor with rCR, overall nor in ER-positive versus ER-negative subgroups. The lack of significant association of sTILs with outcome in the metastatic setting is in line with other retrospective cohort analyses and a post hoc analysis of the MA.31 study [16, 18, 42]. However, post hoc analyses of the CLEOPATRA and PANACEA study showed a positive association of sTILs and OS [15, 43]. In the CLEOPATRA study, all patients received dual HER2-blockade, which is shown to have a synergistic effect on increasing NK-cell migration [15, 44]. The PANACEA study combined trastuzumab with PD1-inhibitor pembrolizumab and showed a better response rate in a subgroup of patients with metastatic samples that harbored at least 5% sTILs. This could suggest that patients with high sTILs in HER2-positive MBC might have unfavorable outcome with conventional anti-HER therapy but may benefit from a combination of anti-HER2 therapy with immune checkpoint inhibitors that can activate cytotoxic T-cells and enhance the anti-tumor immune response [43].

Typical quantitative evaluations of sTILs do not capture different composite immune cell populations that may specifically influence the pro-tumor anti-tumor equilibrium in the tumor microenvironment [10]. For instance, Tregs are a key regulator of the T-cell response. We found a negative association with outcome for Tregs. This finding is in line with several meta-analyses that showed a poor prognosis with high infiltration of Tregs in early breast cancer [45]. It has also been shown that infiltration of Tregs correlates with poor prognostic factors such as ER-negativity, HER2-positivity, lymph node metastasis and high histological grade in early breast cancer [46]. Studies evaluating infiltration of regulatory T-cells in MBC are sparse but confirm a negative association with survival [18]. On a positive note, a recent study in ER-positive metastatic breast cancer showed effective reduction in activated Tregs in the microenvironment after treatment with tamoxifen, pembrolizumab and vorinostat (a histone deacetylase inhibitor), presumably reflecting a remodeling of the tumor microenvironment toward anti-tumor immunity [47].

Recently Sobral-Leite and colleagues showed that infiltration of Tregs is associated with downstream activation of the PI3K pathway [48], a known resistance mechanism in HER2-positive and ER-positive breast cancer. This finding could explain the strong negative association with survival we found in this HER2-positive MBC cohort and provides rationale for evaluating therapies that combine HER2-targeted, PI3K inhibition and Treg-targeted agents. Drugs targeting the PI3K pathway are combined with anti-PD(L)1-targeting drugs in three phase 1 studies for patients with solid tumors with an overactivated PI3K pathway (NCT03673787, NCT03257722, NCT04317105).

Another recent study in breast cancer indicated that the number of Tregs is closely correlated with that of (IL10+) Bregs in TIL aggregates in marginal regions of tumors [49]. Similarly to Tregs, Bregs are negative regulators of anti-tumor immune response and associated with progression of several cancers, including breast cancer [49]. Moreover, B-cells can induce transformation of CD4+ T-cells to Tregs via TGF-β and IL10 and direct contact via the PD1-PDL1 axis in mice models [50, 51]. We did not characterize B-cell subpopulations, therefore we do not know whether Bregs or active B-cells determine the negative association with OS and rCR. Nevertheless, we report a consistent negative impact on outcomes with the infiltration of B-cells. In our cohort less than 1% of patients had a tertiary lymphoid structure (TLS), the prognostic value of TLS was therefore not included in our analyses. We did analyze the spatial colocalization of T-cells and B-cells, which was not associated with outcome (Additional file 4: Results Table). To the best of our knowledge, no study reported a specific role of B-cells in MBC. In early breast cancer, however, infiltration of B-cells is associated with high pathological complete response rates in in several studies [52, 53]. This observation might indicate plasticity of B-cells and a more immune suppressive role of B-cells in metastatic cancer, which deserves further study and possibly evaluation of targeting Bregs in MBC [54].

We also found a negative association between the *exhausted T-cell* signature in primary tumors and outcome. Exhaustion can be seen as a self-preserving transient state of T-cells, induced under chronic stimulation of antigens. Exhausted T-cells can also be recognized by high expression of CTLA-4, LAG-3, PD-1, and TIM-3 [55]. Therefore presence of exhausted T-cells is associated with benefit from PD1- or PDL1-targeted therapy. In our cohort, none of the patients received such therapy, probably explaining the unfavorable association of the exhausted T-cell signature with outcome. PD1 and PDL1 expression in the primary tumor was not associated with outcome in our cohort. Strikingly, we noticed a higher chance of achieving rCR in samples when PD1-positive T-cells were close to PDL1-positive stromal cells or tumor cells in metastatic samples. In general, PDL1 expression is associated with decreased survival in early breast cancer [56]. However, inhibition of PD1/PDL1, as well as other checkpoints expressed by exhausted T-cells, shows high efficacy in reversing the exhaustion and stimulating anti-tumor activity of T-cells [55]. Among patients with HER2-positive MBC, the PANACEA study showed an objective response in 7 of 46 (15%) of the patients with PDL1 expression. In patients without expression of PDL1 no responses were seen [43]. The KATE2 study demonstrated no clinically significant PFS benefit with the addition of atezolizumab (anti-PDL1) to TDM1 (versus placebo + TDM1) in patients not selected for PDL1 expression [57]. Similarly, no significant clinical activity was seen in heavily pre-treated HER2-positive MBC who received durvalumab (anti-PDL1) and trastuzumab in a phase 1 study [58]. Several phase 2 and 3 studies are recruiting patients with HER-positive MBC and will evaluate a combination of trastuzumab with or without pertuzumab with an anti-PD(L)1 inhibitor, summarized by Griguolo [59] and Costa [60]. To the best of our knowledge, no studies are yet evaluating a combination of HER2-targeted therapy and anti-lymphocyte-activation gene 3 (LAG3) or anti-T cell immunoglobulin and mucin domain-containing protein (TIM3) (and anti-PD(L)1), which could also be an appealing strategy to overcome T-cell exhaustion.

Novel immunomodulatory strategies for patients with HER2-positive MBC might focus on NK-cells. In our study, presence of NK-cells in the primary tumor was not associated with outcome, only NK-cells in metastases showed a trend toward more rCR (OR > 10, *P* = 0.054, Additional file 4). This could suggest that there were not enough NK-cells for a meaningful benefit or NK-cells are inhibited by other infiltrating cells [12]. NK-cells can be inhibited via stimulation of the NK inhibitory receptor (NK group 2 member A [NKG2A]) in the context of MHC class I [11]. Monalizumab, which targets NKG2A, has shown benefit in phase 1 and 2 studies in gynecological malignancies [61] and head and neck cancer [11], respectively. Monalizumab in combination with trastuzumab is now being evaluated in the phase 2 MIMOSA study in patients with HER2-positive MBC (NCT04307329).

In general, metastatic samples had significantly lower infiltration of sTILs, especially CD8+ T-cells compared to the primary tumor. Additionally, spatial analyses from the mIF data showed less colocalization of tumor cells and immune cells in the metastases compared to primary tumors. These results are in line with several studies, that have concordantly shown that metastases harbor fewer sTILs compared to their corresponding primary tumors [43, 62, 63]. On top of that, the site of metastases influences the number of sTILs, with lung metastases harboring more sTILs than bone or liver metastases [18, 43]. It should also be noted that sTILs are dynamic and subject to host factors and changes in the tumor as well as treatment.

We used the Morisita-Horn index to evaluate colocalization of immune cells and tumor cells and between different immune cells. Rather than evaluating the nearest neighbor, the Morisita-Horn index evaluates segregation of cells within a neighborhood [39]. We therefore believe this index provides a better representation of the tumor microenvironment. To strengthen our findings, we also used the Spatial Proximity Score ([SPS]; Additional file 2: Fig. S3B) to evaluate impact of nearest neighbor within the tumor immune microenvironment (Additional file 4: Results Table). The associations with outcome of colocalization of Tregs to other T-cells, PD1-positive T-cells to PDL1-positive stroma and tumor was similar using the SPS or the Morisita-Horn index. The Ecoscore, which classifies the tumor microenvironment as more pro-tumor or more anti-tumor (Additional file 2: Fig. S3C) [64], showed no association with outcome in our cohort (Additional file 4: Results Table).

To the best of our knowledge, our study is the largest real-world cohort of patients with HER2-positive MBC in which the tumor immune microenvironment is evaluated in detail. Another strength of our study is the high correlation of the results over the different technologies that we used to evaluate the tumor immune microenvironment. Nevertheless, using a real-world cohort comes with some limitations. First, mIF and IHC biomarkers were assessed using TMAs which may overestimate the expression of biomarkers compared to whole slides, although the TMA results and gene expression analyses largely corresponded [65]. Second, availability and evaluability of tissue created some limitation for thorough evaluation, including a selection of relatively easier sites for biopsies, excluding bone biopsies, which are more common in ER-positive tumors, relatively few (*n* = 26) matched primary tumors and metastatic samples, and some metastatic samples (*n* = 7, 18%) were taken after treatment, which could have induced a bias in less sTILs and maybe proportionally less samples of patients who achieved rCR. Third, not all patients with metachronous MBC (i.e., metastases developed after treatment for the primary tumor), received trastuzumab as neoadjuvant and/or adjuvant therapy and none of the patients received pertuzumab which is now the recommended first line of treatment for HER2-positive MBC. In addition, 23 patients with oligometastases received local treatment for their metastases as well as systemic therapy. The number of patients in subsets is small, which limits our ability to draw definite conclusions in HER2-positive/ER-positive and HER2-positive/ER-negative subgroups. We could not perform separate analyses in the synchronous subgroup, the subgroup with oligometastases or according to different treatment schedules. Despite these limitations, we provide insight in the tumor immune microenvironment that potentially can help designing treatment combinations for patients with HER2-positive MBC with a unique dataset containing long median follow-up of nine years. In addition, we contribute to the scientific community a resource collection of deep, multi-platform, multi-technology immune composition data for clinically well-annotated HER2-positive MBC. Future studies could elaborate on the results showed in this paper, for instance, in depth analysis of intracellular signaling that influences the function of immune cells could further help in designing treatment strategies.

In conclusion, we present insight into the tumor immune microenvironment and its association with outcome in a real-world cohort of patients with HER2-positive MBC. In our cohort, the infiltration of B-cells and regulatory T-cells in the primary tumor microenvironment are associated with unfavorable OS and lower probability of achieving radiological complete remission. These findings provide insight and rationale to further explore a combination of HER2-targeted therapy with targeted immune-modulating therapy to improve durable responses in more patients.

### Supplementary Information

**Additional file 1**: Supplementary Materials and Methods.

**Additional file 2**: Supplementary Figures.

**Additional file 3**: Supplementary Tables.

**Additional file 4**: Results Table.

**Additional file 5**: De-identified clinical data used in this study.

## Data Availability

The datasets supporting the conclusions of this article are included within the article and its additional files. De-identified clinical data used in this study are included as Additional file 5 as well as the molecular and spatial data generated and analyzed during this study. Normalized gene expression data is available via https://console.cloud.google.com/storage/browser/normalized_ngsdata_her2posmbc_nki_steenbruggen. R scripts are available upon request.

## Abbreviations

ADCC: Antibody-dependent cell cytotoxicity
AR: Androgen receptor
ECM: Extracellular matrix
ER: Estrogen receptor
FFPE: Formalin-fixed paraffin-embedded
H&E: Hematoxylin and eosin
HER2: Human epidermal growth factor receptor 2
HR: Hazard ratio
IHC: Immunohistochemistry
IQR: Interquartile range
LAG3: Lymphocyte-activation gene 3
MBC: Metastatic breast cancer
MIF: Multiplex immunofluorescence
NK: Natural killer
NKG2A: NK group 2 member A
OR: Odds ratio
OS: Overall survival
PD1: Programmed death ligand 1
PDL1: Programmed death 1
rCR: Radiological complete remission
SPS: Spatial Proximity Score
sTILs: Stromal tumor infiltrating lymphocytes
TDM-1: Ado-trastuzumab emtansine
TGF-β: Transforming growth factor beta
TIM3: T cell immunoglobulin and mucin domain-containing protein
TMA: Tissue microarray
Tregs: Regulatory T-cells
