# Supplementary material for: B-cells and regulatory T-cells in the microenvironment of HER2+ breast cancer are associated with decreased survival: a real-world analysis of women with HER2+ metastatic breast cancer
Source: Breast Cancer Res. 2023 Oct 4;25:117. doi: 10.1186/s13058-023-01717-1 (PMC10552219; doi:10.1186/s13058-023-01717-1)
Supplement: Supplementary file 1 — Additional file 1: Supplementary Materials and Methods. [file 13058_2023_1717_MOESM1_ESM.docx]

**Additional File 1** Supplementary Materials and Methods to *Steenbruggen et al.* B-cells and regulatory T-cells in the microenvironment of HER2+ breast cancer are associated with decreased survival: a real-world analysis of women with HER2+ metastatic breast cancer

**Supplementary Materials and Methods**

*Next-generation* *RNA sequencing*

RNA was isolated using the Qiagen AllPrep DNA/RNA FFPE Kit (cat. No 80234) using the QiaCube. Samples having sufficient RNA yield (200 ng) were processed with TruSeq RNA Exome Library Prep kit (Illumina) according to the manufacturer’s instructions. Samples were sequenced with 65-bp single-end reads on a HiSeq2500 in high-output mode using V4 chemistry (Illumina). Median sequencing depth was 18.9 million reads. Two samples had too few unique reads for further analysis (less than 0.5 million unique reads) and were excluded. Transcripts were quantified from the reads using Salmon v1.0.0, using GC-bias correction and a k-mer size of 29, against the Gencode v32 reference transcriptome. The transcript-level transcript-per-million values reported by Salmon were summed for each gene to give the final gene expression values. Pre-treatment primary tumor samples were selected for evaluation of expression data (excluding 17 patients with only post-treatment samples).

*70-gene and 80-gene signatures*

70-gene high was defined as index equal or below 0.00 and 70-gene low as index above 0.00. The final 80-gene result was the highest score among the three subtype scores (Luminal-, HER2- and Basal-type index) per sample.(1) By combining the 70-gene and 80-gene results, luminal type breast cancer could be divided into Luminal A (80-gene Luminal and 70-gene low) and Luminal B (80-gene Luminal and 70-gene high).(2,3) In addition to the subtype, we used the continuous 70-gene index and the Luminal-, human epithelial growth factor receptor 2 (HER2)-, and Basal-type score.

*Tissue micro array* (*TMA) construction*

Two independent pathologists screened the H&E slides of all tissue blocks for the infiltration of breast cancer tissue and lymphocytic infiltrate in the tumor. Tissue blocks with sufficient amount of tumor were selected for TMAs preparation. We used three 0.6 mm cores from the primary breast tumor per patient, if available one 0.6 mm core of a pre-treatment biopsy of the primary tumor per patient, and, if available, one 1 mm core from metastases. In total 5 TMAs were constructed, consisting of 205 cores of 135 patients.

*Multispectral immunofluorescence (MIF)*

Freshly-cut four-μm sections from paraffin TMA blocks and tonsil sections were stained on a Discovery Ultra instrument (Ventana). Sections were subjected to sequential rounds of staining with each primary antibody followed by a secondary HRP-conjugated polymer. Signal amplification was achieved with TSA-Opal fluorophores (Akoya Biosciences). In between each round, a heat-induced epitope retrieval (HIER) step was performed to remove primary and secondary antibodies before staining with the next primary antibody. After all sequential antibody stains were complete, the slides were counterstained with spectral DAPI (Akoya Biosciences) and mounted with Vectashield mounting medium (Supplementary Table S2).

Slides were scanned using the Vectra 3.0 slide scanner (PerkinElmer). The tonsil section was used as a positive control for the various markers. TMA cores were annotated using the Phenochart whole slide viewer (PerkinElmer) to scan at high resolution (20x). Image analysis was performed using the inForm automated image analysis software package (Akoya Biosciences) to identify cells, their spatial location, and the expression of the above markers on a cell by cell basis. (4) All cores were manually checked, with supervision of a pathologist, for evaluability to the following criteria: 1) a minimum of 25% of the core is scanned; 2) the core contains at least 200 tumor cells (CK-positive cells); and 3) the segmentation of cells is representative for both the tumor cells and the stromal cells. Cell phenotypes were assigned using auto-thresholding algorithms of mean fluorescence intensity per cell for the individual markers.(5–7) Results for the different cell populations are presented as a percentage of the total cell count within the core image. We and other have previously shown that the staining data are reproducible, reliable, and of high quality.(8,9)

*Spatial distribution analysis (colocalization)*

The spatial relationships of different cells in the tumor microenvironment was also evaluated using the nearest neighbor distance distribution function G(r). G(r) is a spatial metric that represents the probability of finding at least one cell of any given type within a radius (r) of another cell of a given type. This probability distribution can be applied to quantify the relative colocalization of any two types of cells, for example the fraction of tumor cells with at least one T-cell with a given radius (see Supplementary Figure S3B).(8) For this study, we defined a Spatial Proximity Score (SPS_20_) as the area under the G(r) curve where r ranges from 0 to 20μm.

Finally, we calculated an Ecoscore(10) to spatially describe the balance of stromal and immune cells in the TME. Each image was divided into squares of 100 μm and each square was classified as having high or low numbers of stromal cells or TILs relative to the number of tumor cells in that square. An Ecoscore was calculated as the ratio of stroma-low/TIL-high squares to stroma-high/TIL-high plus stroma-high/TIL-low squares. Evaluation of the Ecoscore assumes: 1) stromal cells aid tumor growth while TILs inhibit tumor growth, and 2) high stromal cell density inhibits TIL anti-tumor activity. Therefore, the Ecoscore represents the ratio of anti-tumor niches to pro-tumor niches; a higher value represents a more anti-tumor milieu (see Supplementary Figure S3C). Note that this Ecoscore is the inverse of that calculated by Nawaz et al, where a higher value represents a more pro-tumor microenvironment.(10)

*Immunohistochemistry (IHC) and expression scoring*

FFPE-tissue sections were used for H&E and IHC staining. IHC of TMAs was performed on a BenchMark Ultra autostainer (Roche Ventana Medical Systems). Freshly-cut four-μm sections from paraffin TMA blocks; slides were de-paraffinized by routine techniques and then incubated with the primary antibody. The TMAs were labeled by IHC for androgen receptor (AR), estrogen receptor (ER), progesterone receptor (PR), HER2, HER2 silver in-situ hybridization (sish), CD3, CD8, CD20, CD56, CD68, and programmed death ligand 1 (PDL1). Specific antibody clones, dilutions, and incubation times used are listed in Supplementary Table S2.

Slides were scanned at Leica Aperio ScanScope and uploaded on to the online pathology platform SlideScore (www.slidescore.com). Two pathologists, who were blinded for outcomes, evaluated the stained slides digitally and independently. ER, PR and AR were scored by percentage of nuclear labeling (0-100%). HER2 expression was scored using labeling intensity (0-3+) of proportion of complete membranous staining. HER2 amplification by SISH-probe analysis was scored as: no amplification, low amplification or high amplification. CD3, CD8, CD20, CD56, CD68, and PDL1 were assessed as the percentage of the total stromal area. The level of expression was scored for each TMA core separately, the maximum score per sample (3 cores) was used.

**References Supplementary Materials and Methods**

1. Krijgsman O, Roepman P, Zwart W, Carroll JS, Tian S, de Snoo FA, et al. A diagnostic gene profile for molecular subtyping of breast cancer associated with treatment response. Breast Cancer Res Treat. Netherlands; 2012;133:37–47.

2. Agendia N V. Agendia NV. MammaPrint and BluePrint Breast Cancer Recurrence and Molecular Subtyping Kit - Package Insert. 2020.

3. Mittempergher L, Delahaye LJMJ, Witteveen AT, Spangler JB, Hassenmahomed F, Mee S, et al. MammaPrint and BluePrint Molecular Diagnostics Using Targeted RNA Next-Generation Sequencing Technology. J Mol Diagn. United States; 2019;21:808–23.

4. Stack EC, Wang C, Roman KA, Hoyt CC. Multiplexed immunohistochemistry, imaging, and quantitation: A review, with an assessment of Tyramide signal amplification, multispectral imaging and multiplex analysis. Methods. 2014;70:46–58.

5. Otsu N. A threshold selection method from gray-level histograms. IEEE Trans Syst Man Cybern. 1979;9:62–6.

6. Huang Wang L-K, Wang M-JJ. Image thresholding by mimizing the measures of fuzziness. 1995. page 41–5.

7. Zack GW, Rogers WE, Latt SA. Automatic measurement of sister chromatid exchange frequency. J Histochem Cytochem. 1977;25:741–53.

8. Campbell MJ, Yau C, Bolen J, Vandenberg S, Hoyt C, Brown-Swigart L, et al. Abstract CT003: Analysis of immune cell infiltrates as predictors of response to the checkpoint inhibitor pembrolizumab in the neoadjuvant I-SPY 2 TRIAL. Cancer Res. 2019;79:CT003 LP-CT003.

9. Parra ER, Uraoka N, Jiang M, Cook P, Gibbons D, Forget M-A, et al. Validation of multiplex immunofluorescence panels using multispectral microscopy for immune-profiling of formalin-fixed and paraffin-embedded human tumor tissues. Sci Rep. Nature Publishing Group UK; 2017;7:13380.

10. Nawaz S, Trahearn NA, Heindl A, Banerjee S, Maley CC, Sottoriva A, et al. Analysis of tumour ecological balance reveals resource-dependent adaptive strategies of ovarian cancer. EBioMedicine. Elsevier B.V.; 2019;48:224–35.
