# Supplementary material for: B-cells and regulatory T-cells in the microenvironment of HER2+ breast cancer are associated with decreased survival: a real-world analysis of women with HER2+ metastatic breast cancer
Source: Breast Cancer Res. 2023 Oct 4;25:117. doi: 10.1186/s13058-023-01717-1 (PMC10552219; doi:10.1186/s13058-023-01717-1)
Supplement: Supplementary file 2 — Additional file 2: Supplementary Figures. [file 13058_2023_1717_MOESM2_ESM.docx]

**Additional File 2** Supplementary Figures to *Steenbruggen et al.* B-cells and regulatory T-cells in the microenvironment of HER2+ breast cancer are associated with decreased survival: a real-world analysis of women with HER2+ metastatic breast cancer

**Supplementary Figures**

Figure S1 CONSORT Flow diagram of patients included in the analysis on each evaluation platform

Abbreviations: MBC, metastatic breast cancer; NKI, The Netherlands Cancer Institute.

Figure S2 Example of multiplex immunofluorescent staining

Microphotographs of a representative example of multiplex immunofluorescent staining, showing; nuclei (DAPI, blue). tumor cells (CK+, yellow), PD-L1-positive cells (red), unclassified macrophages (CD68+ cells; (turquoise) and PD1-positive cells (orange) as single gates. CD3-positive cells (green) and CD8-positive cells (magneta) as dual gate and all gates merged. ×200 magnification.

Abbreviations: CK, cytokeratin.

Figure S3 Evaluating the spatial composition of the tumor microenvironment with 3 different methods


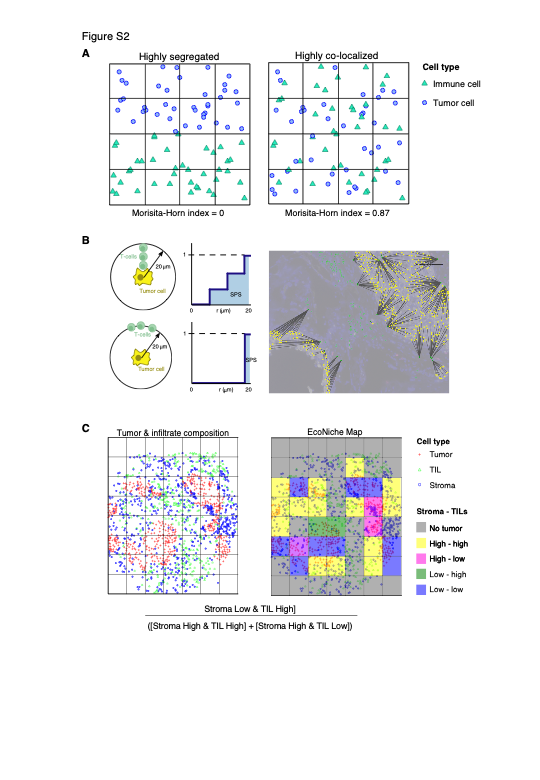


**A** is a graphic explanation of the calculation of the Morisita-Horn index. The Morisita-Horn index is a statistical measure between 0 and 1 of dispersion of individual cells in a “population”, in this case tumor area. Numbers closer to 0 mean highly segregated cell populations. Numbers closer to 1 mean highly co-localized cell populations.

**B** is a graphic explanation of the calculation of the Spatial Proximity Score (SPS). The G(r) function gives the proportion of cell type A (T-cell) within r μm of cell type B (tumor cell). The Spatial Proximity Score is the area under the G(r) curve.

**C** is a graphic explanation of the calculation of the Ecoscore. Each core is divided into “Econiches”, i.e., a square of 100 μm. For each Econiche is calculated whether the Econiche is Stroma Low & TIL High or Stroma High & TIL High or Stroma High & TIL Low. The Ecoscore represents the ratio of anti-tumor niches to pro-tumor niches; a higher value represents a more anti-tumor milieu.

Figure S4 Different technologies are congruent in reporting biology

Clustered heatmap of pairwise biomarker correlation coefficients, annotated by type and platform (rows) and by pathway and constituent cells (columns), using complete linkage. Overarching pathways are indicated by colors on top. Platform can be gene expression (yellow), TMA IHC (orange), or TMA MIF (red). Type refers to whether TMA measurements are of an individual cell type (lavender), proliferating cells (purple), or cell-cell spatial relationships (dark purple).

Abbreviations: ECM, extracellular matrix.

Figure S5 Samples of the primary tumor and metastasis from the same patient cluster together

Unclustered heatmap of all samples and all datapoints included in the analyses. On top clinical characteristics are shown, see legend on the right. Left of the heat map the platform used, type of data and overarching pathway are shown, see legend on the right. Overarching pathways are indicated by colors on top.
Abbreviations: ECM, extracellular matrix; LTS, long-term survivor; MTS, medium-term survivor; rCR, radiological complete response; STS, short-term survivor.

Figure S6 Among classical breast cancer phenotypes, ER- and PR-positivity are associated with better outcome

**A** is a close-up of Figure 3 of all breast cancer subtype related associations with outcomes. All significant associations in at least one analysis are shown in the dot plot. Blue dots indicate increased overall survival or higher likelihood of radiological complete response. Red dots indicate decreased overall survival or lower likelihood of radiological complete response. The size of the dot is proportional to the P value with larger dots indicating a smaller P value. The background color is white for P <0.05, light grey for P >0.05 and <0.10 and grey for P ≥0.10.

**B** shows the overall survival probability according to expression of the *ESR1/PGR* signature in primary tumors split in tertiles. Patients with the highest expression of the *ESR1/PGR* signature have the best overall survival.

**C** shows the distribution of expression of the *ESR1/PGR* signature in primary tumors of patients with no radiological complete response (yellow) and patients with radiological complete response (blue).

Abbreviations: ECM, extracellular matrix; rCR, radiological complete response.

Figure S7 Stromal tumor infiltrating lymphocytes are not associated with outcomes

**A** shows the distribution of stromal tumor infiltrating lymphocytes in primary tumors and different metastases. The highest levels of stromal tumor infiltrating lymphocytes are seen in lung metastases.

**B** shows the overall survival probability according to stromal tumor infiltrating lymphocytes level (low, intermediate, or high). Low was defined as ≤10%, intermediate between 11 and 59% and high as ≥60% stromal tumor infiltrating lymphocytes as measured on whole H&E slides.

**C** shows the distribution of stromal tumor infiltrating lymphocytes in primary tumors of patients with no radiological complete response (yellow) and patients with radiological complete response (blue).

**D** shows the distribution of stromal tumor infiltrating lymphocytes in metastases of patients with no radiological complete response (yellow) and patients with radiological complete response (blue).

Abbreviations: rCR, radiological complete response; STILS, stromal tumor infiltrating lymphocytes.
