# Supplementary material for: B-cells and regulatory T-cells in the microenvironment of HER2+ breast cancer are associated with decreased survival: a real-world analysis of women with HER2+ metastatic breast cancer
Source: Breast Cancer Res. 2023 Oct 4;25:117. doi: 10.1186/s13058-023-01717-1 (PMC10552219; doi:10.1186/s13058-023-01717-1)
Supplement: Supplementary file 3 — Additional file 3: Supplementary Tables. [file 13058_2023_1717_MOESM3_ESM.docx]

**Additional File 3** Supplementary Tables to *Steenbruggen et al.* B-cells and regulatory T-cells in the microenvironment of HER2+ breast cancer are associated with decreased survival: a real-world analysis of women with HER2+ metastatic breast cancer

**Supplementary Tables**

| Table S1. Specification of Signatures | | | | | |
| --- | --- | --- | --- | --- | --- |
| Immune Signature | **Method of calculation** | **Cancer type** | **Signature Functional Category** | **Genes** | **PubMed ID** |
| B_cells | mean | Non-specifc | B-cell | BLK, CD19, FCRL2, MS4A1, KIAA0125, TNFRSF17, TCL1A, SPIB, PNOC | 28239471 |
| Bcell_mg_IGJ | mean | Breast | B-cell | POU2AF1, CD38, IGLJ3, IGHM, IGHG3, ICAP-1A, IGHG3, IGKC, KIAA0746, IGJ, LOC91316, IGL@, IGKV1D-13, MGC27165, PACAP | 18593943 |
| CD8_CD68_ratio | ratio | Breast | Macrophage: T/B-cell ratio | CD8A, CD68 | 22039576 |
| Chang_core_serum | median | Breast | core serum response | CEP78, LSM3, LRRC40, STK17A, RPN1, JUNB, NUP85, FLNC, HMGN2, RPP40, UQCR10, AIMP2, CHEK1, VTA1, EXOSC8, CENPO, PNO1, SLC16A1, WDR77, UBE2J1, NOP16, NUDT1, SMC2, SLC25A5, NUPL1, DLEU2, PDAP1, CCBL2, COX17, BCCIP, PLG, RGS8, SNRPC, PLK4, NUTF2, LSM4, SMS, EBNA1BP2, C13orf27, VDAC1, PSMD14, MYCBP, SMURF2, GNG11, F3, IL7R, BRIP1, HNRNPA2B1, DCK, ALKBH7, HN1L, MSN, TPM1, HYLS1, HAUS1, NUP93, SNRPE, ITGA6, CENPN, C11orf24, GGH, PFKP, FARSA, EIF2AK1, CENPW, TUBA4A, TRA2B, UMPS, MRTO4, NUDT15, PGM2, DBNDD1, SNRPB, MNAT1, NUP35, TCEB1, HSPB11, C19orf48, ID3, IPO4, FARSB, EIF4G1, SKA1, MFSD11, PLAUR, MARVELD2, MCM3, DHFR, RNF41, ID2, H2AFZ, CDK2, NCLN, ZWILCH, DYNLT1, C16orf61, SLC25A40, RHOC, CCT5, PDIA4, SNRPA, RBM14, PDLIM7, PITPNC1, TPM3, CORO1C, ERLIN1, PAICS, TPRKB, SKA2, MYBL1, SH3BP5L, BRCA2, SAR1A, POLR3K, MRPS28, NUP107, TUBG1, PNN, FAM167A, RFC3, MYL6, MCM7, MAGOHB, FAM89B, TOMM40, CDCA4, MT3, MTHFD1, PSMD12, MYBL2, CKLF, NRIP3, EZR, C12orf24, GPLD1, SRM, RAB3B, NLN, MT1F, TNFRSF12A, TPI1, HAS2, APOO, FBXO41, MRPL37, GSTCD, SDC1, WDR54, RNF138, APITD1, RMND5B, ENO1, MAP3K8, TMEM130, SNX17, KRR1, TAGLN, PA2G4, RUVBL1, SNRPD1, LOXL2, POLE2, MAPRE1, IMP4, EMP2, PSMD2, MET, IFRD2, LMNB2, PLOD2, NCEH1, NME1, STRA13, ACTL6A, DLEU1, SNRPA1, CBX1, LYAR, PTPLB, PFN1, CENPJ, COTL1, SPRYD7, USPL1, MRPL12, ADAMTS1, GLRX3, WSB2, MRPS16, DCLRE1B, MKKS, C3orf26, CPEB4, SPAG17, MLF1IP, UAP1, COQ2, WDHD1, DCBLD2, KIAA0090, SAR1B, PSMA7, PSMC3, COPS6, DUT, PPIH, PHF19, TPM2, MCTS1, EIF4EBP1, HNRNPR | 15701700 |
| Dendritic cells | average | Non-specifc | Dendritic cell | CCL13, CD209, HSD11B1 | 28239471 |
| ECM1 | correlation to centroid | Breast | Extracellular matrix |  | 18044827 |
| ECM2 | correlation to centroid | Breast | Extracellular matrix |  | 18044827 |
| ECM3 | correlation to centroid | Breast | Extracellular matrix |  | 18044827 |
| ECM4 | correlation to centroid | Breast | Extracellular matrix |  | 18044827 |
| ERBB2_amplicon | mean | Breast | ERBB2 | ERRB2, STARD3, PRODH, GRB7 | 24516633 |
| ESR1_PGR | mean | Non-specifc | ESR1/PGR | ESR1, PGR |  |
| Exhausted Tc Cells | mean | Non-specifc | T-cell | LAG3, CD244, EOMES, PTGER4 | 28239471 |
| ICS5_score | weighted mean | Breast |  | CXCL13, CLIC5, HLA-F, TNFRSF17, XCL2 | 24172169 |
| IR7_score | weighted mean | Breast |  | C1QA, IGLC2, LY9, TNFRSF17, SPP1, XCL2, HLA-F | 17683518 |
| Macrophages | mean | Non-specifc | Macrophage | CD68, CD84, CD163, MS4A4A | 28239471 |
| Mast cells | mean | Non-specifc | Mast cell | TPSB2, TPSAB1, CPA3, MS4A2, HDC | 28239471 |
| MCD3_CD8 | median | Breast | T/B-cell | SRPX, IGFBP6, ENPP2, SEMA3G, CIDEA, GPX3, GPD1, CD36, RBP4, AOC3, LPL, FABP4, ADIPOQ, PLIN1, ADH1B, FHL1, LEP, CD34, SPRY1, PROS1, PPAP2A, AKAP12, JAM3, NDN, SPARCL1, ITM2A, AQP1, C7, TSPAN7, JAM2, CAV1, GNG11, LDB2, LHFP, CDH5, ABCA8, MEOX1, DARC, VWF, CLDN5, ERG, SLIT2, PECAM1, CDO1, CFD | 21214954 |
| MHC-I | median | Breast | T/B-cell | HLA-G, HLA-A, HLA-B, HLA-C, HLA-F | 19272155 |
| MHC-II | median | Breast | T/B-cell | CTSS, CD74, HLA-DRB1, HLA-DQA1, HLA-DMB, HLA-DPA1, HLA-DPB1, HLA-DMA, HLA-DRA, LCP2, SRGN, PTPRC | 19272155 |
| Mod10_ECM | PC | Breast | Extracellular matrix |  | 24516633 |
| Mod11_proliferation | PC | Breast | Proliferation | CDKN3, NDC80, RNASEH2A, CENPA, SMC2, CENPE, RAD51AP1, PLK4, NMU, KIF2C, TMSB15A, UBE2C, CHEK1, ZWINT, OIP5, CRABP1, ECT2, EIF4EBP1, EZH2, FEN1, HSPA4L, TPX2, FOXM1, NCAPH, PRAME, PDSS1, KIF4A, RAD54B, ASPM, FBXO5, ATAD2, RACGAP1, GPSM2, DONSON, HMMR, BIRC5, KIF11, LMNB1, MAD2L1, MCM4, MCM5, MKI67, MMP1, MYBL1, MYBL2, NEK2, NUSAP1, GTSE1, GINS2, PLK1, FAM64A, ERCC6L, NCAPG2, CEP55, FANCI, HJURP, MCM10, DEPDC1, C1orf112, CENPN, PBK, KIF15, CIAPIN1, ACTR3B, GPR126, SPC25, RAD21, RFC3, RFC4, RRM2, NCAPG, STIL, SKP2, SOX11, SQLE, AURKA, TAF2, TARS, BUB1B, TK1, TMPO, TOP2A, PHLDA2, TTK, LRP8, DSCC1, MLF1IP, E2F8, SHCBP1, SLC7A5, ANP32E, KIF18A, CDC7, CDC45, RAD54L, TTF2, PIR, ACTL6A, GGH, CCNA2, CCNB1, PRC1, CCNB2, CCNE2, EXO1, AURKB, PTTG1, TRIP13, KIF23, APOBEC3B, MTFR1, ESPL1, DLGAP5, CDK1, MELK, GINS1, CDC6, CDC20, NCAPD2, KIF14 | 24516633 |
| Mod3_IFN | PC | Breast | Interferon | IFI44, IFI44L, DDX58, IFI6, IFI27, IFIT2, IFIT1, IFIT3, CXCL10, MX1, OAS1, OAS2, OAS3, HERC5, SAMD9, HERC6, DDX60, RTP4, IFIH1, STAT1, TAP1, OASL, RSAD2, ISG15 | 24516633 |
| Mod4_immune_T/Bcell | PC | Breast | T/B-cell | CD96, CD52, SEMA4D, CXCL13, SP140, CCR7, CTSW, DOCK2, EVI2B, FCN1, KLRK1, FLI1, PLCL2, FYB, IPCEF1, PPP1R16B, CCDC69, STAP1, GPR18, ICOS, GPR171, GZMA, GZMB, GZMK, IGJ, IL2RB, IL2RG, IL7R, ITGA4, ITK, KLRB1, LCK, LGALS2, LRMP, LTB, SH2D1A, CXCL9, NCF4, GIMAP6, IL21R, TRAT1, PLAC8, UBASH3A, POU2AF1, RHOF, LAX1, BANK1, SIRPG, PRF1, DOCK10, PRKCB, CRTAM, PTGDS, PTPRC, PTPRCAP, TNFRSF17, CCL19, SELL, BCL11B, SLAMF1, TNFRSF1B, CCR2, TRAF3IP3, TCL1A, VNN2, PSTPIP1, CD2, CD3G, CD247, CD7, CD8A, CD19, MS4A1, CD27, AIM2, CD37, CYTIP, CD69, CD79A, FAM65B, KIAA0125, P2RY14 | 24516633 |
| Mod5_immune_TcelBcell | PC | Breast | T/B-cell | IGSF6, LILRB2, BTN3A3, UBD, CXCL13, GNLY, CXCR6, CTSC, HCP5, PIM2, SP140, CCR7, CTSS, CYBB, FCN1, TFEC, SEL1L3, FYB, GBP1, LAMP3, ADAMDEC1, GPR18, ICOS, GPR171, GZMH, GZMB, GZMK, BIRC3, IFNG, IL2RG, IL15, IDO1, CXCL10, IRF1, ISG20, ITK, LAG3, LCK, LYN, CXCL9, NKG7, TRAT1, MGC29506, PLAC8, POU2AF1, CRTAM, SLAMF8, PSMB9, PTPN7, SLAMF7, BCL2A1, TNFRSF17, CCL5, CCL8, CCL13, CCL18, CCL19, CXCL11, SELL, SAMSN1, RTP4, CLEC7A, TAP1, WARS, PLA2G7, ZBED2, NPL, RUNX3, VNN2, CD3G, IL32, CD8B, CD19, CD86, AIM2, CD38, CYTIP, LOC96610, CD69, CD79A | 24516633 |
| Mod8_histone | PC | Breast |  |  | 24516633 |
| Mod8_stromal/Mixed | PC | Breast | Stroma |  | 24516633 |
| NHI_5gene | mean | Breast | Immune-dominated prognostic | IGK@, GBP1, STAT1, IGLL5, OCLN | 21479927 |
| PD1_data | value | -- | Single gene immune therapy targets | PDCD1 | -- |
| PDL1_data | value | -- | Single gene immune therapy targets | CD274 | -- |
| TGFB_score | mean | Breast | TGF-beta | MMP3, MARCKSL1, IGF2R, LAMB1, SPARC, FN1, ITGA4, SMO, MMP19, ITGB8, ITGA5, NID1, TIMP1, SEMA3F, RHOQ, CTNNB1, MMP2, SERPINE1, EPHB2, COL16A1, EPHA2, TNC, JUP, ITGA3, TCF7L2, COL3A1, CDH6, WNT2B, ADAM9, DSP, HSPG2, ARHGAP1, ITGB5, IGFBP5, ARHGDIA, LRP1, IGFBP2, CTNNA1, LRRC17, MMP14, NEO1, EFNA5, ITGB3, EPHB3, CD44, IGFBP4, TNFRSF1A, RAC1, PXN, PLAT, COL8A1, WNT8B, IGFBP3, RHOA, EPHB4, MMP1, PAK1, MTA1, THBS2, CSPG2, MMP17, CD59, DVL3, RHOB, COL6A3, NOTCH2, BSG, MMP11, COL1A2, ZYX, RND3, THBS1, RHOG, ICAM1, LAMA4, DVL1, PAK2, ITGB2, COL6A1, FGD1 | 21050467 |
| TILs | mean | Non-specifc | T/B-cell | PTPRC | 28239471 |
| Tregs | mean | Non-specifc | T/B-cell | FOXP3 | 28239471 |

Table S2. Specification of Antibodies

| **Marker** | **Clone** | **Clonality** | **Source** | **Scoring** |
| --- | --- | --- | --- | --- |
| Antibodies used in multiplex immunofluorescence panels | | | | |
| CD3 | 2GV6 | Rabbit monoclonal | Roche Ventana | % of total count |
| CD8 | 4B11 | Mouse monoclonal | Leica | % of total count |
| CD20 | L26 | Mouse monoclonal | Roche Ventana | % of total count |
| CD68 | PG-M1 | Mouse monoclonal | DAKO | % of total count |
| CD117 |  | Rabbit polyclonal | DAKO | % of total count |
| FOXP3 | SP97 | Rabbit monoclonal | Spring Bioscience | % of total count |
| PD1 | EPR4877 | Rabbit monoclonal | Abcam | % of total count |
| PDL1 | E1L3N | Rabbit monoclonal | Cell Signaling | % of total count |
| CK | AE1/AE3 | Mouse monoclonal | DAKO | % of total count |
| KI-67 | 30-9 | Rabbit monoclonal | Roche Ventana | % of total count |
| Antibodies used in single immunohistochemistry | | | | |
| ER | SP1 | Rabbit monoclonal | Roche Ventana | % nuclear staining |
| PR | 1 E 2 | Rabbit monoclonal | Roche Ventana | % nuclear staining |
| AR | SP107 | Rabbit monoclonal | Spring / ITK | % nuclear staining |
| HER2 IHC | 4B5 | Rabbit monoclonal | Roche Ventana | intensity membranous staining |
| HER2 sish | HER2 DNA Probe | - | Roche Ventana | number of copies |
| CD3 | SP7 | Rabbit monoclonal | Thermo Scientific | % of infiltrate |
| CD8 | C8/144B | Mouse monoclonal | Agilent (DAKO) | % of infiltrate |
| CD20 | L26 | Mouse monoclonal | Agilent (DAKO) | % of infiltrate |
| CD56 | MRQ-42 | Rabbit monoclonal | Cell Marque | % of infiltrate |
| CD68 | KP1 | Mouse monoclonal | Agilent (DAKO) | % of infiltrate |
| PD-L1 | 22C3 | Mouse monoclonal | Agilent (DAKO) | % of infiltrate |

| Table S3. Stromal TIL percentages are different according to the interval between primary tumor and metastases | | | | | | |
| --- | --- | --- | --- | --- | --- | --- |
|  | **number of primary tumor samples with evaluable TILs** | **median sTIL value (IQR) primary tumors** | **P value^a^** | **number of metastases samples with evaluable TILs** | **median sTIL value (IQR) metastases** | **P value^a^** |
|  | n=109 |  |  | n=39 |  |  |
| Age at diagnosis MBC, years |  |  | 0.664 |  |  | 0.100 |
| ≤ 50 years | 52 | 7.50  (1.75-30.00) |  | 21 | 1.00  (1.00-5.00) |  |
| > 50 years | 57 | 7.00  (3.00-30.00) |  | 18 | 4.00  (1.00-13.75) |  |
| Interval between primary tumor and MBC | | | 0.020 |  |  | 0.538 |
| De novo MBC | 26 | 5.00  (1.00-9.25) |  | 7 | 1.00  (1.00-3.00) |  |
| ≤ 36 months | 41 | 8.00  (3.00-30.00) |  | 12 | 2.50  (1.00-10.00) |  |
| > 36 months | 42 | 10.00  (5.00-37.50) |  | 20 | 2.00  (1.00-5.50) |  |
| HER2 IHC |  |  | 0.748 |  |  | 0.178 |
| 1+ | 7 | 10.00  (4.00-15.00) |  | 4 | 1.00  (1.00-7.00) |  |
| 2+ | 7 | 15.00  (5.00-40.00) |  | 3 | 1.00  (1.00-1.00) |  |
| 3+ | 79 | 7.00  (2.00-30.00) |  | 19 | 4.00  (1.00-12.50) |  |
| HER2 SISH |  |  | 0.552 |  |  | 0.542 |
| negative | 5 | 7.00  (5.00-20.00) |  | 0 | - |  |
| Low amplification | 8 | 1.00  (1.00-25.00) |  | 2 | 5.00  (2.50-7.50) |  |
| High amplification | 58 | 8.50  (3.00-30.00) |  | 11 | 1.0  (3.00-10.00) |  |
| ER-status at diagnosis |  |  | 0.268 |  |  | 0.760 |
| ER-positive | 64 | 7.00  (1.00-20.00) |  | 20 | 1.00  (1.00-5.00) |  |
| ER-negative | 45 | 7.00  (5.00-40.00) |  | 17 | 4.00  (1.00-10.00) |  |
| PR-status at diagnosis |  |  | 0.745 |  |  | 0.059 |
| PR-positive | 36 | 10.00  (2.75-30.00) |  | 11 | 1.00  (1.00-2.00) |  |
| PR-negative | 73 | 7.00  (3.00-30.00) |  | 26 | 4.00  (1.00-13.75) |  |
| AR-status |  |  | 0.521 |  |  | 0.742 |
| AR-positive | 83 | 10.00 (3.50-30.00) |  | 25 | 1.00  (1.00-7.00) |  |
| AR-negative | 15 | 5.00  (1.50-35.00) |  | 13 | 4.00  (1.00-5.00) |  |
| Grade primary tumor |  |  | 0.172 |  |  | 0.558 |
| grade 1 or 2 | 21 | 7.00  (1.00-15.00) |  | 10 | 2.50  (1.00-4.75) |  |
| grade 3 | 75 | 10.00  (3.00-32.50) |  | 19 | 3.00  (3.00-15.00) |  |
| Oligometastases (≤ 3 metastases) | |  | 0.578 |  |  | 0.747 |
| Oligometastases | 35 | 7.00  (2.50-22.50) |  | 15 | 1.00  (1.00-6.00) |  |
| multiple metastases | 74 | 7.00  (3.00-30.00) |  | 24 | 2.00  (1.00-6.25) |  |

^a^ P values are based on Mann Whitney U test or Kruskal-Wallis test, whatever test was appropriate for the data.

Abbreviations: IQR, interquartile range; MBC, metastatic breast cancer; sTIL, stromal tumor infiltrating lymphocytes.
